# Cytological analysis of hybrids among triticales and trigopiros

**DOI:** 10.1590/S1415-47572009005000070

**Published:** 2009-12-01

**Authors:** Maia Fradkin, Eduardo Greizerstein, Héctor Paccapelo, Víctor Ferreira, Ezequiel Grassi, Lidia Poggio, María Rosa Ferrari

**Affiliations:** 1 Laboratorio de Citogenética y Evolución, Departamento de Ecología, Genética y Evolución, Facultad de Ciencias Exactas y Naturales, Universidad de Buenos Aires, Ciudad Autónoma de Buenos AiresArgentina; 2 Facultad de Agronomía, Universidad Nacional de La Pampa, Santa Rosa, La PampaArgentina; 3 Genética, Facultad de Agronomía y Veterinaria, Universidad Nacional de Río Cuarto, Río Cuarto, CórdobaArgentina; 4 Física Biológica, Facultad de Ciencias Veterinarias, Universidad de Buenos Aires, Ciudad Autónoma de Buenos AiresArgentina

**Keywords:** hybrids, meiosis, tricepiro, univalents, reductional division

## Abstract

We studied three different tricepiros: (Don Santiago x Don Noé), (Cumé x Horovitz) and (Cumé x Don Noé). The tricepiro (Don Santiago x Don Noé) was obtained by crossing the triticale Don Santiago INTA (AABBRR, 2n = 6x = 42) with the trigopiro Don Noé INTA (AABBDDJJ, 2n = 8x = 56). The number of chromosomes for the F_1_ was 2n = 49, the most frequent meiotic configuration being 14 bivalents and 21 univalents. The univalents were situated in the periphery of the equatorial plane, whereas the bivalents were located in the central zone. The chromatids in some of the univalents split when bivalents underwent reductional division in anaphase I. There were few laggard chromosomes or chromatids at this phase. The number of chromosomes (2n = 48-58) was high and variable, and the number of bivalents per cell (18-23) also high in F _3_ individuals. In all F _8_ tricepiros (Don Santiago x Don Noé), F _12_ tricepiros (Cumé x Horovitz) and F _12_ tricepiros (Cumé x Don Noé), the number of chromosomes (2n = 42) was the same, these retaining the rye genome, as demonstrated by GISH and FISH. These new synthesized allopolyploids constitute interesting models for investigating the evolutionary changes responsible for diploidization, and the chromosomal and genomic re-ordering that cannot be revealed in natural allopolyploids.

## Introduction

Polyploidy has played an important role in plant speciation. It has been estimated that perhaps more than 70% of Angiosperms have undergone polyploidy one or more times at some point in their evolutionary history (Ma and Gustafson, 2005). Molecular and cytological studies have revealed that many species traditionally considered as diploid, such as maize, *Arabidopsis* and soybean, among others, are in fact paleopolyploids (Ma *et al.*, 2004).

A segmental allopolyploid originated from related species is highly likely to undergo homoeologous chromosome pairing during meiosis, thus reducing its fertility. Moreover, the coexistence of genetically closely related genomes results in genome-wide gene redundancy, which also contributes to the genetic instability of allopolyploids (Feldman and Levy, 2005).

Recent studies have shown that rapid genome changes occur following F_1_ or allopolyploid formation: 1) the non-random elimination of coding and non-coding DNA sequences; 2) epigenetic changes, such as DNA methylation of coding and non-coding DNA, leading, among other things, to gene silencing; 3) the activation of genes and retro-elements, which, in turn, alters the expression of adjacent genes (Feldman and Levy, 2005); and 4) chromosome reorganization, with the gain or loss of chromosomes or whole genomes (Bernardo *et al.*, 1988; Soler *et al.*, 1990; Soltis and Soltis, 2000; Ferrari *et al.*, 2005). An example of a particular case of the harmonic coexistence of several genomes achieved by chromosomal rearrangement and gain or loss of chromosomes or even completed genomes, was observed in the tricepiro Don René INTA (Ferrari *et al.*, 2005). This cultivar was obtained by crossing a hexaploid triticale (2n = 42, AABBRR, A, B from wheat and R from rye) with an octoploid trigopiro (2n = 8x = 56, AABBDDJJ, with A, B, D from wheat and J from wheatgrass), and became stable at 2n = 42*. In situ* hybridization techniques (GISH and FISH) have demonstrated that the genomic constitution of this allopolyploid is AABBRR with introgression of *Thinopyrum* (J genome) in chromosome pair 6 of the wheat A genome (Ferrari *et al.*, 2005).

Tricepiros are synthetic cereals of high forage value obtained by crossing wheat, rye and wheatgrass. They have a marked resistance to disease and high tolerance to freezing and drought (Ferreira *et al.*, 2007).

The aim of this work was to analyse the meiotic behaviour of a tricepiro Don Santiago x Don Noé F_1_ hybrid. On the other hand, our purpose was to determine the chromosome number and genomic composition in advanced generations of tricepiros from different origins (triticale Don Santiago x trigopiro Don Noé, triticale Cumé-UNRC x trigopiro Horovitz and triticale Cumé-UNRC x trigopiro Don Noé), so as to improve knowledge on the mechanisms of polyploid stabilization.

## Materials and Methods

###  Plant material

*Triticale Cumé-UNRC* was obtained and cultivated at the Universidad Nacional de Río Cuarto (UNRC), Province of Córdoba, Argentina.

*Tricepiro (Don Santiago x Don Noé*): The F_1_ hybrid was obtained at the Universidad Nacional de La Pampa, Province of La Pampa, Argentina, by crossing triticale Don Santiago INTA with trigopiro Don Noé INTA. Successive generations were obtained by self-fertilization, all of which being cultivated at the same locale. The F_1_, F_3_ and F_8_ generations were those studied in this work.

*Tricepiro (Cumé x Horovitz)* was obtained at the Universidad Nacional de Río Cuarto (UNRC), Province of Córdoba, Argentina, by crossing triticale Cumé UNRC with trigopiro“Horovitz”. Successive generations were obtained by self-fertilization, all being cultivated at the same locale. F_12_ individuals were those studied in the present work.

*Tricepiro (Cumé x Don Noé)* was obtained at the Universidad Nacional de Río Cuarto (UNRC), Province of Córdoba, Argentina, by crossing triticale Cumé-UNRC with trigopiro Don Noé INTA. Successive generations were obtained by self-fertilization, all being cultivated at the same locale. F_12_ individuals were hereby studied.

All trigeneric hybrids had triticale as the female parent and trigopiro as the pollen donor.

Between three and five individuals were studied in all the generations, except in F_1_, in which only one individual was studied.

###  Meiotic analysis

For meiotic analysis, young flowers were fixed in 3:1 (absolute alcohol: acetic acid) and kept at -5 °C. Anthers were squashed in 2% propionic haematoxylin with 1% ferric citrate as mordant (Nuñez, 1968). The Meiotic Index (MI) was calculated as follows: MI = (Number of normal tetrads/ Total number of tetrads studied) x 100.

### *In situ* hybridization

*In situ* hybridization techniques (GISH, genomic *in situ* hybridization, and FISH, fluorescent *in situ* hybridization) were performed on mitotic cells according to Ferrari *et al.* (2005). GISH was carried out using rye genomic DNA as a probe and unlabelled wheat DNA for blocking. Rye genomic DNA was isolated from adult leaves of *S*. *cereale cv. Quehue* and wheat genomic DNA from *T. aestivum cv. Klein Estrella.*

FISH was carried out using the pSc 119.2 probe. This probe contains the 120-bp family subclone isolated by McIntyre *et al.* (1990), and permits the identification of all the rye chromosomes and some of the wheat.

At least 10 cells per plant of each hybrid were analyzed.

## Results

###  Tricepiro (Don Santiago x Don Noé)

**Figure 1:**
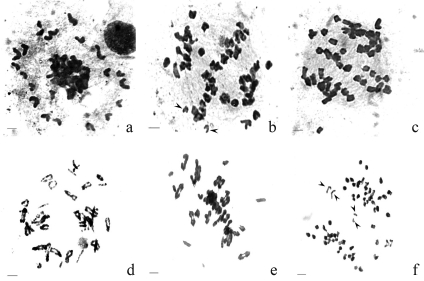
(a-c) Meiotic cells of F_1_ tricepiro (Don Santiago x Don Noé). (a) Metaphase I with 21 univalents in the periphery of the equatorial plate; (b and c) early anaphase I, some chromosomes are a the poles while others remain in the equatorial plate. Note the presence of 49 chromosomes in (c). (d-f) Meiotic cells of F_3_ hybrid tricepiro (Don Santiago x Don Noé) with different chromosome numbers. (d) Diplotene with at least 20 bivalents; (e) Metaphase I cell with ca 23 bivalents; (f) anaphase I with 58 chromosomes. Arrows indicate univalents separating chromatids. Bars = 10 μm.

The presence of 21 univalents in the periphery of the equatorial plate in 70% of metaphase 1 cells (n = 16) was detected in F_1_ individuals. The remaining chromosomes were very close to each other, and appeared to be bivalents (Figure 1a). In 50% of the cells in anaphase I, 14 chromosomes were observed at each pole, while the rest remained in the equatorial plate (Figure 1b,c). Figure 1c shows the presence of 49 chromosomes in the F_1_ hybrid. Some univalents disjointed the chromatids at this phase (Figure 1b). Most of the univalents and chromatids were located at the poles at the end of anaphase I. At telophase I (n = 33), 36% of the dyads were normal, whereas 33% showed only one lagging chromosome and 31% more than one lagged and up to eight lagging.

The F_3_ segregants were obtained by self-fertilization. When meiosis was analysed in seven individuals from F_3_, we found a high number of bivalents per cell (from 18 to 23), besides a chromosome number varying from ca 2n = 48 to 58 (Figure 1d,e). A few lagging chromosomes with precocious separation of sister chromatids were observed at anaphase I (Figure 1f). The tetrads were analysed, the MIs so obtained ranging between 7.3 and 53.0. The MI was also determined in F_8_, the final value being 51.0.

**Figure 2:**
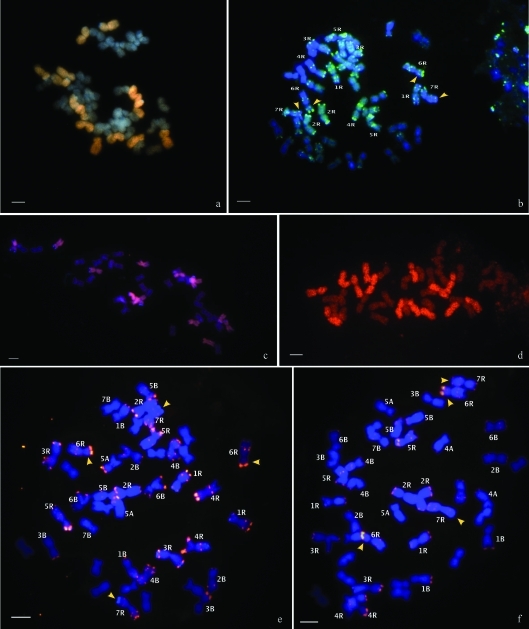
(a) Mitotic metaphase cells of two F_8_ tricepiros (Don Santiago x Don Noé). GISH using DNA from *S. cereale* as a probe, detected with Cy3, blocked with unlabelled wheat DNA and with DAPI counterstaining. Note that the chromosome number is 2n = 42 and the 14 rye chromosomes disclose strong hybridization signals. b) FISH using pSc119.2 probe, detected with digoxigenin and revealed with FITC (green) in a mitotic triticale Cumé cell. (c and e) Mitotic metaphase cells of tricepiro (Cumé x Horovitz) and (d and f) of tricepiro (Cumé x Don Noé). (c and d) GISH using DNA from *S. cereale* as a probe, detected with Cy3 and blocked with unlabelled wheat DNA. Note that the chromosome number is 2n = 42 and the 14 rye chromosomes disclose strong hybridization signals. (e and f) FISH using the pSc 119.2 probe and detected with conjugate Streptavidine-Cy3 (red). Arrows show hybridization signals in 6RS, and in 7RL chromosomes of triticale Cumé and tricepiro (Cumé x Don Noé), absent in tricepiro (Cumé x Horovitz). Bars = 10 μm.

GISH was carried out in two F_8_ lines by using rye DNA as a probe and wheat DNA as a blocking, and by counterstaining with DAPI. This study revealed a chromosome number 2n = 42 with 14 rye chromosomes (Figure 2a).

###  Triticale Cumé

This cultivar is a hexaploid triticale (2n = 42). Seven pairs of rye chromosomes were identified with the pSc119.2 probe (Figure 2b).

###  Tricepiro (Cumé x Horovitz)

This hybrid was analysed at an advanced generation, namely F_12_, and was found to possess a chromosome number of 2n = 42. *In situ* hybridization with rye genomic DNA, blocking with wheat genomic DNA and counterstaining with DAPI led to the detection of 14 rye chromosomes (Figure 2c).

We were able to identify 14 rye chromosomes (Figure 2e) with the pSc119.2 probe. The signals observed with this probe in the middle of the 6RS chromosome and at the terminal end of the 7RL chromosome of the parental triticale Cumé were absent in this hybrid (Figure 2b,e). This probe also permitted the identification of the 14 chromosomes of the wheat B genome and various chromosomes of the A genome (Figure 2e).

###  Tricepiro (Cumé x Don Noé)

This hybrid was analysed in F_12_ and was found to have a chromosome number of 2n = 42. As with all the other hybrids, *in situ* hybridization with rye genomic DNA, blocking with wheat genomic DNA and counterstaining with DAPI led to the detection of 14 rye chromosomes (Figure 2d).

With the pSc119.2 probe, we were also able to identify the 14 rye chromosomes and the 14 chromosomes belonging to the B genome, besides some chromosomes from the wheat A genome (Figure 2f). The signal pattern of the hydrid rye chromosomes seems to be similar to that observed in its progenitor, triticale Cumé (Figure 2b).

## Discussion

F_1_ of the tricepiro hybrid (Don Santiago x Don Noé) has a chromosome number of 2n = 49. Since its parents were a hexaploid triticale (2n = 6x = 42, AABBRR) and an octoploid trigopiro (2n = 8x = 56, AABBDDJJ), the expected genomic composition of F_1_ would be AABBDRJ. In this F_1,_ 14 bivalents and 21 univalents were observed in metaphase I. Theoretically, it is expected that the bivalents correspond to homologous pairing between chromosomes belonging to wheat A and B genomes, whereas the 21 univalents could be chromosomes of D (*Triticum aestivum)*, R (*Secale cereale*) and J *(Thinophyrum ponticum*) genomes. The low frequency of pairing between wheat A, B and D genomes due to the presence of the Ph gene, together with the low homology of R and J genomes with each other and with wheat genomes, as observed by certain authors (Dewey, 1984; Jauhar *et al.*, 2004), support this hypothesis.

Meiotic analysis of F_1_ of the tricepiro (Don Santiago x Don Noé) infer that both early segregated bivalents and the 14 chromosomes observed at each pole of anaphase I could belong to A and B genomes. Equational segregation in anaphase I was observed in some univalents. This phenomenon was also observed by other authors in anaphase I of hybrids in which wheat and rye were involved (Gorban and Shulyndin, 1978; Silkova *et al.*, 2003). Silkova *et al.* (2003) postulated that the split of univalents could be part of the mechanism responsible for meiotic restitution in wheat-rye polyhaploids. In the present work, equational segregation observed in anaphase I could be involved in the increase in the number of chromosomes and bivalents present in F_3_ individuals.

The chromosome number of the tricepiro F_8_ (Don Santiago x Don Noé) lines, as well as the F_12_ (Cumé x Horovitz) and (Cumé x Don Noé) lines, was 2n = 42. GISH revealed the presence of 14 rye chromosomes in all. In previous studies, crossing different accesions of triticales and trigopiros gave rise to the same chromosome number (2n = 42) in advanced generations (Ferrari *et al.*, 2005, Ferreira *et al.*, 2007). These results and those obtained in the present work showed similar behaviour in triticale x trigopiro hybrids, since all became stabilized with 2n = 42 and retained the rye R genome. The same tendency was observed in different hybrids involving wheat (Bernardo *et al.*, 1988, Soler *et al.*, 1990). Bernardo *et al.* (1988) studied rye chromosome behaviour in the progeny from AABBR hybrids, and proposed that there was total elimination of rye chromosomes when individually present but retention when the complete genome was present.

The presence of univalents that undergo chromatid separation in anaphase I, and the low frequency of lagging chromosomes in telophase I, as observed in the F_1_ of tricepiro (Don Santiago x Don Noé), together with the high number of bivalents in the F_3_ individuals, led us to suggest preferential migration of the R genome. A mechanism of gamete selection that promotes the retention of the R genome could also be associated with this behaviour.

It is interesting to point out the difference found between the tricepiros (Cumé x Horovitz) and (Cumé x Don Noé), concerning hybridization signals of the pSc119.2 probe in 6RS and 7RL chromosomes, when compared with their progenitor triticale Cumé. The loss of these signals in the tricepiro (Cumé x Horovitz) could be a consequence of the role of fast change mechanisms on the structure and composition of the genome, as postulated by certain authors (Ma *et al.*, 2004; Ma and Gustafson, 2005; Feldman and Levy, 2005; Ferrari *et al.*, 2005).

These new synthesized allopolyploids will be interesting models for investigating those evolutionary changes that are responsible for genetic diploidization and chromosomal and genomic reordering that cannot be revealed in natural allopolyploids.
